# GENOTOXICITY AND CYTOTOXICITY OF X-RAYS IN CHILDREN EXPOSED TO PANORAMIC RADIOGRAPHY

**DOI:** 10.1590/1984-0462/;2017;35;3;00010

**Published:** 2017-07-20

**Authors:** Evelyn Louise Antonio, Aguinaldo José do Nascimento, Antonio Adilson Soares de Lima, Maria Suely Soares Leonart, Ângela Fernandes

**Affiliations:** a Prefeitura Municipal de Curitiba, Curitiba, PR, Brasil.; b Universidade Federal do Paraná (UFPR), Curitiba, PR, Brasil.

**Keywords:** Radiation induced abnormalities, Genotoxicity, Children

## Abstract

**Objective:**

To assess the genotoxicity and cytotoxicity produced by X-rays in the epithelium of the oral mucosa of infants exposed to panoramic radiography.

**Methods:**

The sample consisted of 30 healthy children, 19 females and 11 males, ranging in age from 4 to 10 years (average of 7 years of age). Oral mucosa cells were collected by liquid-based cytology immediately before and after seven days following the exposure to panoramic radiography. Smears were processed and stained using the modified Feulgen Rossenbeck technique. Bud and broken egg nuclear projections, changes in the form of micronuclei, and genotoxic and cytotoxic changes of pyknosis, karyorrhexis and karyolysis were analyzed and quantified.

**Results:**

The frequency of pyknosis, buds and broken eggs was significantly higher after exposure to X-rays (p<0.05), but there was no statistically significant difference regarding gender, as well as in the other changes studied.

**Conclusions:**

Exposure to X-rays emitted during submission to panoramic radiography may induce cell death in the epithelium of children’s oral mucosa. No evidence was found for a significant genotoxic effect.

## RESUMO

**Objetivo:**

Analisar a genotoxicidade e a citotoxicidade produzidas por raios X no epitélio da mucosa oral de crianças durante a obtenção da radiografia panorâmica.

**Métodos:**

A amostra foi constituída por 30 crianças saudáveis, sendo 19 do sexo feminino e 11 do masculino, com faixa etária de 4 a 10 anos (média de 7 anos de idade). As células epiteliais da mucosa oral foram coletadas por meio de citologia esfoliativa em base líquida imediatamente antes e após sete dias da obtenção da radiografia panorâmica. Os esfregaços foram processados e corados utilizando a técnica de *Feulgen Rossenbeck* modificada. Foram analisadas e quantificadas projeções nucleares dos tipos *bud* e *broken egg*, alterações genotóxicas na forma de micronúcleo e alterações citotóxicas dos tipos picnose, cariólise e cariorrexe.

**Resultados:**

A frequência de picnose, *bud* e *broken egg* foi significativamente maior após a exposição aos raios X (*p*<0,05), porém não houve diferença estatisticamente significante em relação ao sexo, bem como nas demais alterações estudadas.

**Conclusões:**

A exposição aos raios X emitidos durante a obtenção da radiografia panorâmica pode induzir à morte celular no epitélio da mucosa oral de crianças. Não foi encontrado indício significativo de efeito genotóxico.

## INTRODUCTION

Ionizing radiation can induce cytotoxicity,^1^ ^,^ ^2^ ^,^ ^3^ ^,^ ^4^ ^,^ ^5^ ^,^ ^6^ ^,^ ^7^ ^,^ ^8^ ^,^ ^9^genotoxicity^4^ and carcinogenesis^10^ ^,^ ^11^ in human tissues. Low doses of radiation, such as those emitted during panoramic radiography,^12^ are capable of causing deleterious and cumulative biological effects on living organisms. Thus, the use of diagnostic methods that use ionizing radiation should have a well-grounded clinical indication,^13^ ^,^ ^14^ since X-rays have a deleterious effect on epithelial cells. In addition, some studies report that children may be more susceptible to the harmful effects of X-rays when compared to adults.^14^ ^,^ ^15^ ^,^ ^16^

Genetic alterations, such as micronuclei formations and nuclear aberrations, are initial biological effects of carcinogenesis.^2^ Therefore, studies on the genotoxic effects induced by X-rays in the epithelium are important in order to identify the risk of cancer development and to act in its prevention,^17^ since the biomonitoring of the patients by means of exfoliative cytology allows the possibility of verifying and accompanying the presence of cellular atypia before the occurrence of neoplastic clinical manifestations.

Micronuclei are fragments of chromosomes or whole chromosomes that were lost during cell mitosis because of a clastogenic event - which causes chromosomal breakage - or an aneugenic event - which interferes with the mitotic spindle.^18^ X-rays are clastogenic agents that induce the formation of micronuclei, in addition to other nuclear alterations. The frequency of micronuclei is used as a parameter to verify the degree of exposure and extent of damage caused to the DNA by an environmental agent, functioning as a biomarker, and indicating the individual’s susceptibility to the development of cancer.^19^ However, the micronucleus test has its specificity increased when it registers cellular degenerative changes that indicate cell death,^1^ ^,^ ^4^ such as pycnose, karyorrex and karyolysis, and the bud and broken egg nuclear projections.

This study aimed to verify if the X-rays emitted during the panoramic radiography can induce the increase in frequency of micronucleus, picnosis, karyorrhexis, karyolysis, bud and broken egg in the oral mucosa epithelium of children.

## METHOD

This work was approved by the Research Ethics Committee of the Health Sciences Department of the Universidade Federal do Paraná (UFPR) under protocol number 761.096.09.07. Oral mucosa cells were obtained from 30 healthy children - 11 males and 19 females -, ranging in age from 4 to 10 years - with an average of 7 years of age -, who were referred by the pediatric dentistry clinic of UFPR to perform panoramic radiography. The legal guardians for the children authorized the study by signing an Informed Consent.

The legal guardians completed a questionnaire with the following data about the child: age, gender, history of previous exposure to X-radiation, use of mouthwash containing alcohol, use of medications, presence of systemic diseases or alterations that compromise the oral mucosa. The children were authorized to participate in the study by their legal guardians. They were up to ten years of age, of both sexes, and were included in the sample. Children above 10 years of age - for hormonal reasons - and/or who had one of the following conditions observed for less than 28 days were excluded from the sample: submission to ionizing radiation; use of mouthwash containing alcohol; presence of mucosal changes; use of medication or the manifestation of a disease capable of interfering with the cell nucleus. Previous exposure to these genotoxic factors would increase the frequency of nuclear alterations during cell turnover, which occurs every 7 to 28 days. Panoramic radiographs were performed at the Dental Radiology Service of UFPR in a Siemens Orthophos CD model extra-oral radiographic machine with 60 Kv, 16 mA and 14.1 s.

The collection of mature epithelial cells from children’s oral mucosa was performed immediately prior to the panoramic radiograph and seven days^17^ after its completion. The right side of the jugal mucosa was slightly scraped with five clockwise movements and gentle manual pressure, using a cylindrical cytological brush (Cervical Brusch®, Kolplast, São Paulo, Brazil), after rinsing the mouth with running water. Cells were stored in a Falcon tube containing 1 mL of methanol/acetic acid in the ratio of 3:1, centrifuged at 130 x g for 5 minutes, fixed and deposited on clean slides. After drying, the slides were stained by the modified Feulgen Rossenbeck method.^20^ The preparation of the slides was performed in a standardized way^9^ ^,^ ^18^ by a single technician in a single day.

**Figure 1:**
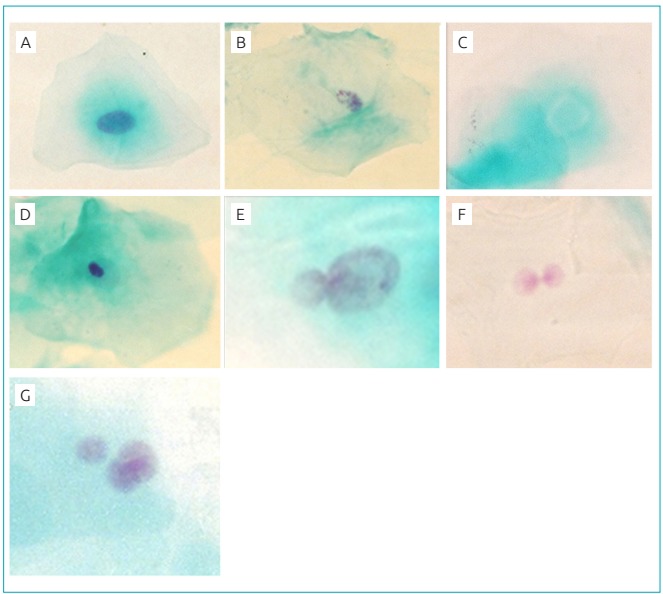
Nuclear alterations analyzed: (A) normal nucleus; (B) karyorrhexis; (C) karyolysis; (D) picnosis; (E) bud; (F) broken egg; (G) micronucleus.

The slides, after hiding their identifications, were analyzed under a light microscope with a magnitude of 400x, and the alterations found were confirmed in 1000x. The micronuclei were analyzed according to the criteria established by Sarto et al.^21^ as a parameter of genotoxicity. The criteria described by Tolbert et al.^17^ were used for the analysis of broken egg, bud, pycnose, karynx and caryolysis alterations. For each slide, a thousand viable cells^18^ were analyzed by a single experienced observer. The cellular alterations analyzed are shown in Figure 1.

For the comparison of means, Student’s *t*-test was used for paired samples. Statistical significance was set at *p*<0.05.

## RESULTS

The frequency of cells that presented micronuclei and other alterations before and seven days after panoramic radiography is shown in Table 1. The frequency of broken egg (p=0.005), bud (p=0.006) and picnosis (p=0.039) was significantly higher after exposure to X-rays. No significant differences were observed regarding the gender of the participants.

**Table 1:**
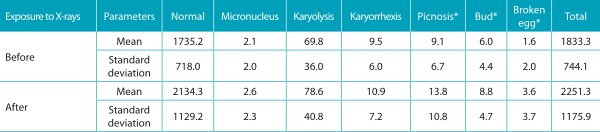
Frequency of nuclear alterations in children exposed to panoramic radiography.

*Statistically significant differences - Student’s *t*-test for paired samples, *p*<0.05. Data expressed in ‰.

## DISCUSSION

In general, children are more susceptible to toxic agents than adults,^22^ so ionizing radiation can be a mutagenic agent with cumulative actions.^23^ Considering that an individual may be repeatedly subjected to x-rays over a lifetime, the effect of successive X-ray exposures and repetitions should be considered, as there may be an increase in the frequency of nuclear alterations following such events.^3^ Although the radiation dose received during panoramic radiography is low,^15^ children are more sensitive to radiation than adults.^24^ Thus, the cumulative effect of small doses on sensitive tissues could trigger cytotoxic effects, resulting in chronic cellular aggression, compensatory cell proliferation, tumor development and carcinogenesis.^2^

Despite the aforementioned considerations, Ribeiro et al.^6^ have shown an increase in similar cytotoxic alterations in children and adults submitted to panoramic radiography. The authors concluded that children were no more susceptible to X-ray agents when compared to adults. In this study, the sample size and types of nuclear alterations analyzed were amplified in relation to the studies of Ribeiro et al.^6^ and Angelieri et al.,^2^ observing the effects of cytotoxicity after exposure to X-rays and also a significant increase of other nuclear degenerative alterations: bud and broken egg, which are different from those found by these authors. Therefore, further studies on the effects of X-rays on children are needed, observing their short- and long-term implications, since the risk of damage from low doses of radiation in this population is not fully understood.

According to Silva et al.,^3^ female hormones provoke alterations in the epithelial cells of the oral mucosa of women. However, in this study, no gender difference was observed, probably because the age group selected had not passed puberty.

Several authors have demonstrated that exposure to low doses of radiation, such as those emitted during panoramic radiography, causes cytotoxicity in tissues of the oral mucosa,^1^ ^,^ ^2^ ^,^ ^3^ ^,^ ^4^ ^,^ ^5^ ^,^ ^6^ ^,^ ^7^ ^,^ ^8^ ^,^ ^9^but does not cause an increase in the frequency of micronuclei.^1^ ^,^ ^2^ ^,^ ^3^ ^,^ ^4^ ^,^ ^5^ ^,^ ^6^ ^,^ ^7^ ^,^ ^8^ ^,^ ^9^ ^,^ ^19^ These data are similar to those observed in this study. However, Cerqueira et al.^4^ found a high frequency of micronuclei in cells of the gingival epithelium after radiation exposure, associating this finding with the fact that the gingival cells were directly affected by X-rays during the panoramic radiography. However, the epithelium of the buccal mucosa is also directly affected by X-rays, but no significant increase in micronucleus frequency was observed after X-ray exposure in this and other similar studies. Perhaps the masticatory, coating and specialized mucosa react differently to the action of X-rays^4^ ^,^ ^7^ and studies are necessary to make this comparison feasible.

The formation of micronuclei is dose dependent and varies depending on the type of radiation used and the radiosensitivity of the involved tissue. However, low doses of X-radiation are capable of inducing DNA breakage, but do not necessarily result in micronuclei.^25^ In this study, the amount of micronuclei and other changes observed were higher after exposure to X-rays compared to epithelial cells before exposure, but were not statistically significant. It is possible that the fact that cytotoxic events decrease cell viability, causing its death by apoptosis, is related to the low frequency of the micronucleated cells found.^1^ ^,^ ^2^

This study demonstrated a significant increase in the frequency of bud, broken egg and picnosis in oral mucosa epithelial cells of children submitted to panoramic radiography, a result similar to Silva et al.^3^ The meaning of buds and broken eggs is still obscure,^26^ and may be related to the normal process of cell division,^1^ ^,^ ^4^ with amplified DNA removed from the nucleus during the S-phase of the cell cycle^27^ or as precursor structures of the micronucleus stage.^28^ ^,^ ^29^ The latter hypothesis would imply genotoxicity of X-rays on the oral mucosa of children. For the moment, it is only possible to state that X-radiation causes genetic instability. Pynotic changes are frequent findings in superficial squamous cells and indicate cell degeneration by intense maturation or early aging linked to a strong inflammatory process, causing the death of the affected cells.

Cell death by apoptosis can occur due to the large amount of lesions caused to the cell’s DNA, making it functionally infeasible to the body. The successive occurrence of these events may delay the renewal of the epithelium lining of the mouth. If the regeneration capacity of the organism is supplanted, degenerative phenomena can cause changes in the epithelium, increasing the predisposition to malignant transformation.^30^ Thus, it is convenient to analyze the X-ray toxicity by means of longitudinal studies to determine if the damage generated is punctual and transient or incorporated and maintained throughout cell divisions.^31^

The limitation of this study is the fact that the microscopic analysis of the nuclear alterations was carried out by a single experienced observer. Despite the application of the intra-examiner calibration and the careful analysis of the slides, it is advisable to compare the inter-examiner data, as recommended in the protocol described by Thomas et al.^23^

Panoramic radiography is considered the first choice exam for the evaluation of children age over five years in dentistry because it allows a wide observation of the buccomaxillofacial complex and exposes the child to a lower dose of X-radiation compared to an intraoral radiographic exam.^14^ However, it should be indicated only when necessary, using an accurate radiographic technique and following the current radioprotection criteria, in order to avoid unnecessary repetition.^3^ ^,^ ^14^ These recommendations can be extrapolated to all exams that use ionizing radiation as a complementary diagnostic method. The results obtained suggest that X-rays emitted during panoramic radiography induce changes in the oral mucosa epithelial cells of children. Therefore, in indicating this imaging examination, professionals should consider the risk of promoting chromosomal changes in the epithelium at each exposure.
